# Supplementary material for: A unified form of low-energy nodal electronic interactions in hole-doped cuprate superconductors
Source: Nat Commun. 2019 Dec 16;10:5737. doi: 10.1038/s41467-019-13497-4 (PMC6914777; doi:10.1038/s41467-019-13497-4)
Supplement: Supplementary file 1 — Supplementary Information [file 41467_2019_13497_MOESM1_ESM.pdf]

A Unified Form of Low-Energy Nodal Electronic Interactions in Hole Doped Cuprate Superconductors

Supplementary Information

T.J. Reber[1*,†],, X. Zhou[1,*], N.C. Plumb[1†],, S. Parham[1],J.A. Waugh[1], Y. Cao[1], Z. Sun[1, †],, H. Li[1], Q. Wang[1], J.S. Wen[2], Z.J. Xu[2], G. Gu[2], Y. Yoshida[3], H. Eisaki[3], G.B. Arnold[1], D. S. Dessau[1*]

1 Dept. of Physics, University of Colorado, Boulder, 80309-0390, USA
2 Condensed Matter Physics and Materials Science Department, Brookhaven National Labs, Upton, New York, 11973 USA
3 AIST Tsukuba Central 2, 1-1-1 Umezono, Tsukuba, Ibaraki 3058568, Japan

[†] Present addresses:

(T. J .R) Dept. of Chemistry, University of Georgia, Athens, GA, 30602;

(N. C. P) Swiss Light Source, Paul Scherrer Institut, CH-5232 Villigen PSI, Switzerland;

(Z. S.) University of Science and Technology of China, Hefei, China.

* Correspondence to: ted.reber@gmail.com,
xiaoqing.zhou@colorado.edu
dessau@colorado.edu

Supplementary Methods.

All data presented here are from high quality $Bi_2Sr_2CaCu_2O_{8+\delta}$ (Bi2212) single crystals cleaved in ultra-high vacuum. The doping level and T_C were confirmed by magnetometry for each sample. We made use of the high energy and momentum resolution of low energy ARPES (1), using 7 eV photons as our probe. This gives a total experimental resolution (10-90% of low temperature Fermi edges) of approximately 4 meV. To be certain the measured intensity is truly representative of the electron population we correct for detector inhomogeneity and nonlinearity (2). All other techniques for collecting ARPES data are standard to the field.

Supplementary Note 1: Pseudogaps and doping phase diagrams from the literature.

A great deal of effort has been put into studies of pseudogapping, which is an incomplete suppression of low-energy spectral weight. In the doping phase diagram of the cuprates, this weight suppression extends up to the temperature range T^* which is well above the superconducting T_c, especially for underdoped sample (supplementary fig. 1b) [3]. Similar to the d-wave superconducting gap, this weight suppression is strongest at the antinodal (π,0) portions of the Brillouin zone, leading to many arguments that the pseudogapping was a type of superconductive prepairing. However, a variety of more recent experiments have indicated that while there are in fact prepairing fluctuations, these don't exist all the way up to T^*, as indicated by the orange shaded regions of supplementary fig. 1a and 1b [4,5,6]. This is consistent with the statements that the antinodal weight suppression pseudogap is not related to prepairing but is instead due to some sort of competing order [7,8].

Interestingly, a very similar T^* scale as observed in the weight suppression pseudogaps is also observed in the transport experiments (supplementary fig. 1a) [9]. In transport, this scale is typically determined as a break from the Marginal Fermi liquid linearity, such as is shown in supplementary fig. 4d of the main paper. This break in linearity has long been associated with the spectral weight pseudogap, partly because of the similar temperature scales and partly from an intuitive argument related to the idea of superconductive prepairing. In particular, it has been argued that prepairing superconducting fluctuations would partially gap out certain scattering channels, dropping the resistivity below the expected linear behavior [10]. This explanation of the connection to the pseudogap is however weakened by the recent findings that the prepairing and T^* scales as measured from gapping experiments are different (supplementary fig. 1). Additionally, from transport it is typically found that the departure from linearity dips into and below the superconducting dome, possibly all the way to zero temperature, signaling a possible quantum critical point (supplementary fig. 1a). And while the two versions of the doping phase diagram are quite similar, seldom if ever has it been asked if the two T^* lines (from spectral weight pseudogapping and from a departure from linearity) may

be due to totally different phenomena, with unfortunate identical names. Our data does indicate that these two phase diagrams are measuring different things, even from the identical ARPES data sets: spectral weight gapping in supplementary fig. 1b and electronic scattering rates in supplementary fig. 1a.

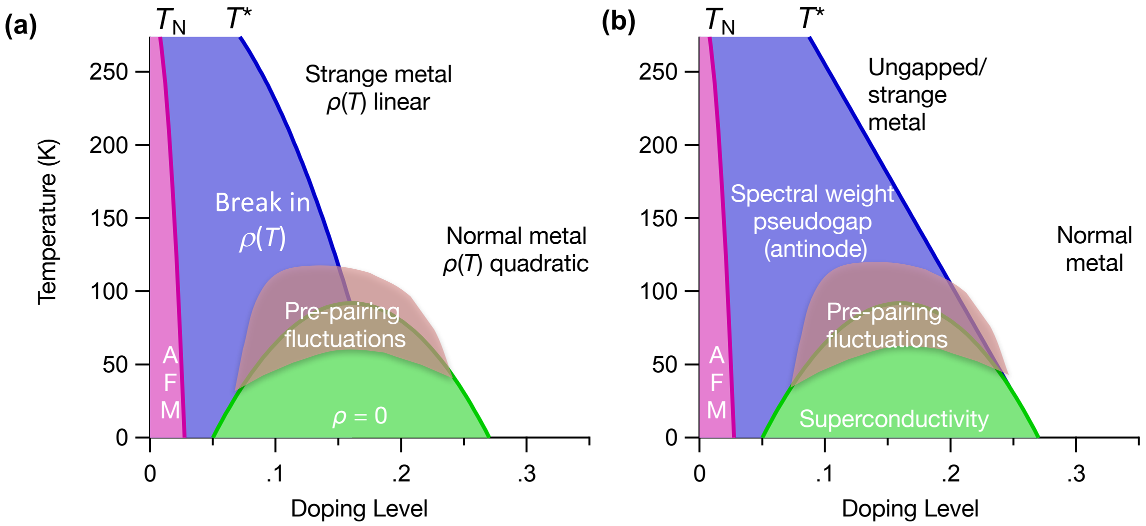

Figure 1. Two popular versions of the doping phase diagram of p-type cuprates (a) as determined by transport (r=resistivity) [ref 7] and specific heat [ref 11] and (b) as determined from probes of gaps and bosonic modes [ref 12]. The T^* line in both are are at similar temperatures and are typically both ascribed to pseudogap physics, though T^* in (b) is not directly a gap measurement.

Supplementary Note 2: Electron self-energies from MDCs

ARPES directly measures the electron spectral function, $A(k,\omega)$and is typically written as:

$$A(k,\omega)=-\frac{1}{\pi}\frac{\Sigma''}{[\omega-\varepsilon_k-\Sigma']^2+[\Sigma'']^2} \tag{1}$$

Assuming a linear bareband, i. e. $\varepsilon_k = v_{Bare}(k-k_F)$, which is in reasonable proximity to E_F, it can be rewritten as

$$A(k,\omega)=-\frac{1/v_{Bare}}{\pi}\frac{\Sigma''(\omega)/v_{Bare}}{\left[k-\left(k_F+\frac{\Sigma'(\omega)-\omega}{v_{Bare}}\right)\right]^2+\left[\frac{\Sigma''(\omega)}{v_{Bare}}\right]^2} \tag{2}$$

In an MDC, ω is fixed and the $A(k,\omega)$ follows a Lorentzian form:

$$Lor(k)=\frac{A}{\pi}\frac{\Gamma/2}{(k-k_0)^2+(\Gamma/2)^2} \tag{3}$$

Consequently the imaginary part of the self energy $\Sigma''(\omega)$is directly proportional to the Lorentzian FWHM, Γ_{MDC}:

$$\Sigma''(\omega)=v_{Bare}*\Gamma_{MDC}(\omega)/2. \tag{4}$$

The bare velocity is constrained by Kramers-Kronig consistency, in which the real part of the self-energy

$$\Sigma'(\omega) = -\frac{1}{\pi}\int \frac{\Sigma''(\omega\prime)}{\omega' - \omega} d\omega' \qquad (5)$$

renormalizes the band velocity. The renormalized band dispersion (i.e. adding $\Sigma'(\omega)$ to the bare dispersion) should match the measured dispersions at all temperatures, under which condition we found a solution v_{Bare}=2.9 eV Å. Please note that while some literature values for the bare velocity differ slightly from our chosen value, even a significant alteration would make almost no difference in the main physics results presented here since the bare velocity enters through a simple linear scaling and hence does not change the dynamical behavior (curvature changes as a function of ω and T).

Supplementary Note 3: ARPES Dispersion kinks

The analysis in this paper principally looks at the scaling of the imaginary self-energy $\Sigma''(\omega,T)$, but it is important to note that real part of the self-energy $\Sigma'(\omega,T)$ can be obtained from $\Sigma''(\omega,T)$ via the Kramer's-Kronig relations. Figure S2 shows the PLL $\Sigma''(\omega,T)$ for many different α parameters as calculated at T=1K as well as the real self-energy $\Sigma'(\omega,T)$ obtained by Kramer's-Kronig of the $\Sigma''(\omega,T)$ data. Aside α=1, all $\Sigma'(\omega,T)$ curves display some curvature in the range plotted, with increasing strength of curvature as α is decreased. Such curvature in $\Sigma'(\omega,T)$ would bring a corresponding curvature to the band dispersion over the same energy range. This would mimic a kink-like dispersion, even in the absence of coupling of the low energy electrons to any low energy mode such as phonons or magnons.

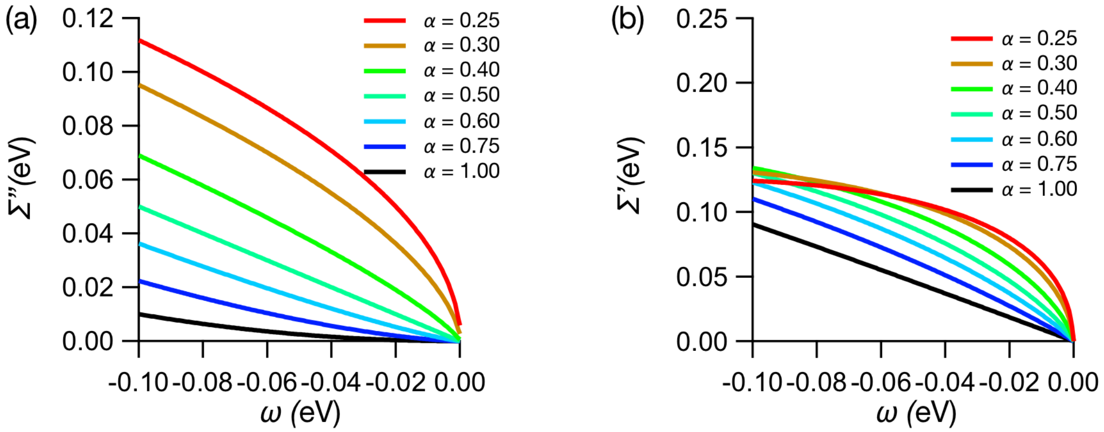

Figure 2. Calculation of the imaginary part a) and real part b) of the power law liquid self-energy Σ at T=1 K for various parameters a. Note that the curve in Σ' will cause a kink-like band dispersion in the energy range where the curvature is observed, i.e. 0-70 meV.

Supplementary Note 4: ω and T scaling from optics

Only a few previous works have tried to test the ω,T scaling for any material, most of them from optics. A detailed work is in ref [11] for the case of Fermi liquids. Here they write the optical scattering rate as:

$$\frac{\hbar}{\tau_{Optical}(\omega)} = \frac{2}{3\pi k_B T_0}\left[(\hbar\omega)^2 + (p\pi k_B T)^2\right] \tag{6}$$

where p is a number that is expected to be 2 for the perfect Fermi Liquid, though it would be 1 for the Fermi Liquid in the single particle scattering rate (ARPES). This equation is essentially equivalent to our equation 1 with α=1 (Fermi liquid), β=π, and the prefactor with $1/T_0$ serving the purpose of our coupling constant λ. As discussed in ref [11] p=2 has not yet been observed in simple metals, and in several correlated metals p has ranged from 1 to 2.4. In their work on underdoped cuprates, Mirzaei et al. have found p=1.5 [26].

Supplementary Note 5: Inhomogeneous broadening, offset parameters, and forward scattering.

When one relates the ARPES data to transport properties, using the full MDC width generally over-predicts the resistivity[12,13,14]. It has been argued[12,13] that a part of the MDC width, possibly due to elastic scattering by impurities, should not be included as a contribution to the transport self-energy.

The cuprates are known to be relatively dirty, with large numbers of impurities added to dope extra carriers into the materials. These impurities might vary from sample to sample, and might scatter the electrons giving an additional peak broadening beyond the intrinsic dynamic ω and T-dependent effects that we are most interested in.

Therefore, we characterize this extra peak-broadening in the typical way, as an ω independent (static) offset to the widths. In equation 1 of the main text we call this offset parameter Γ_0. Here we further extend this definition by breaking Γ_0 up into two components – a forward scattering component Γ_{0FS} and a backwards-scattering component Γ_{0BS}.

$$\Sigma''_{PLL} = \Gamma_{0FS} + \Gamma_{0BS} + \lambda\frac{\left[(\hbar\omega)^2 + (\beta k_B T)^2\right]^{\alpha}}{(\hbar\omega_N)^{2\alpha-1}} \tag{7}$$

It is expected that only the backscattering components should affect the resistivity (through a $\cos\theta$ term where θ is the scattering angle), while both the forward and backscattering components should affect the ARPES MDC widths.

The red curve of supplementary fig. 3a show the doping dependence of the full offset parameter $\Gamma_0 = \Gamma_{0FS} + \Gamma_{0BS}$ obtained from the fits shown in supplementary fig. 1 of the main paper. Γ_0 ranges from about 8 meV for optimal samples to 35 meV for the most heavily underdoped sample. The increase in Γ_0 with underdoping is consistent with the general findings across many spectroscopies that underdoped samples have a greater amount of scattering or peak-broadening.

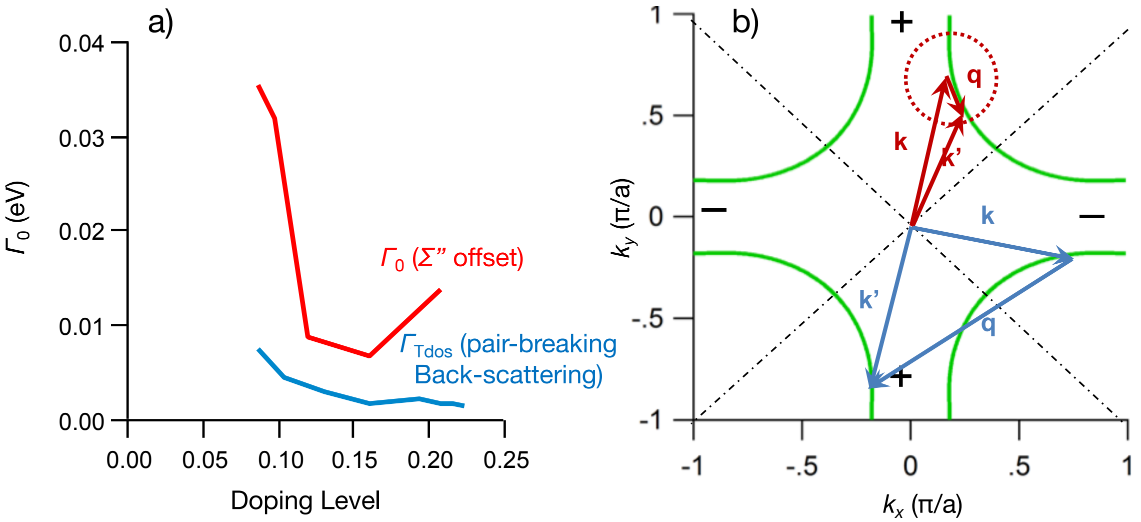

Figure 3 (a) Forward scattering is dominated by out-of-plane disorder and is non pair breaking because it does not flip the phase of the order parameter from + to -. This affects EDC and MDC widths but not the TDoS pair-breaking width. Large angle or back scattering is shown in blue and is pair breaking. It may be thermally created or may be from static impurities in the plane, such as Cu defects. (b) Forward (red) and backwards (blue) scattering contributions to Γ_0.

If we calculate the resistivity using the extracted PLL parameters and the Boltzmann model including the full Γ_0 (see supplementary notes 6), we get the results shown in supplementary fig. 4a, which has a poor agreement with the measured resistivity of main manuscript fig. 4d, while as shown in supplementary fig. 4b an excellent agreement is obtained if we set Γ_0 to zero for the calculations. This confirms that the full Γ_0 plays a role in broadening the ARPES spectra but has only a minimal role in transport.

An estimate for Γ_{0BS} comes from the new Tomographic Density of States (TDoS) method of ARPES analysis, in which we found that the pair-breaking scattering rates in the superconducting state were up to an order of magnitude smaller than what is determined directly from the MDC widths [15,16]. Because forward or small-q scattering will mostly not switch the phase of the d-wave order parameter it is not expected to break the pairs, while back scattering can change the phase and break the pairs (supplementary fig. 3b). In supplementary fig. 3a we show the extracted Γ_{TDoS} as a function of doping (blue) which we relate to Γ_{0BS}. We use these values of Γ_{0BS} in supplementary fig. 4c. The difference between including no scattering offset (supplementary fig. 4b) and including Γ_{0BS} is subtle enough that we can not use these curves to distinguish. This is why we set all offset terms to zero for calculating resistivity in the main paper.

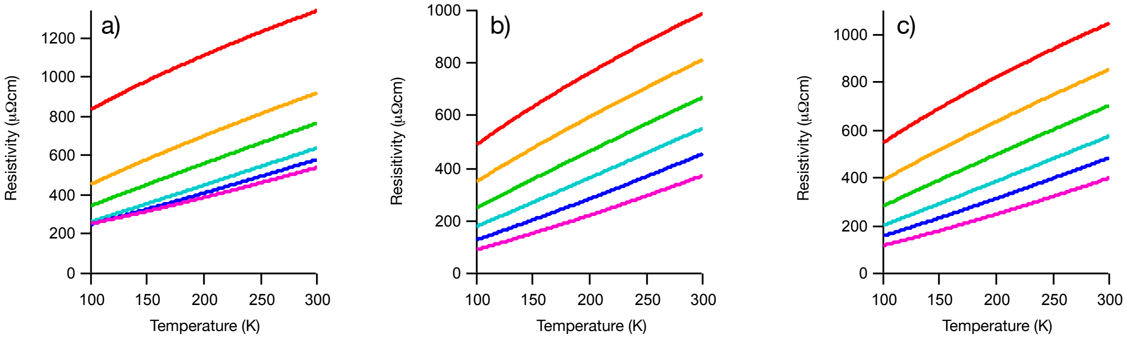

Figure 4. Boltzmann transport calculations with a) the full Γ_0 included; b) no Γ_0 included and c) the pair-breaking scattering rate Γ_{TDoS} [15] or back-scattering term Γ_{0BS} included. The full Γ_0 appears not to contribute to the conductivity. A large part of it is likely forward scattering from out-of-plane impurities which does not contribute to the transport self-energy.

Supplementary Note 6. Formulae and parameters for calculating the resistivity using the Drude and Boltzmann formulas

Here we describe two simple methods for calculating the temperature dependent electrical resistivity, with the primary input being the extracted electronic scattering rates as a function of temperature (the PLL form of the self energy). Other inputs are Fermi surfaces and electronic band dispersions or band masses.

A very simplistic approximation is the Drude model which allows us to convert the electron self energy data into electrical resistivity as follows:

$$\sigma(T) = \frac{\mathrm{ne}^2\tau(T)}{m} \tag{8}$$

where $\hbar/\tau(T)$ is twice of $\Sigma''(\omega=0,T)$ and m is the band mass ~ 1.08 mass of the electron obtained as $\hbar k_F/v_{\mathrm{bare}}$ along the nodal direction, n is the nominal carrier density or doping level p (0.16 * 9*10^{21} Cu sites cm^{-3} for optimal doping), and e is the electronic charge. As shown in main manuscript fig. 4c, resistivity calculated from this simplistic model agrees very well with the measured resistivity. In particular, with ω=0 the resistivity follows a power law temperature dependence $\rho\sim T^{2\alpha}$, which has been independently observed in overdoped cuprates[28].

To address more details on the impact of Fermi surface topology to the transport properties, we use the Boltzmann conductivity tensor to convert the ω=0 electronic self-energy data of main manuscript fig. 4a into electrical conductivity (inverse of resistivity) (main manuscript fig. 4b) using the following equation [17],

$$\sigma = e^2\int\frac{d^3k}{4\pi^3}\tau(k)v(k)v(k)\left[-\frac{\partial f}{\partial\varepsilon}\right]_{\varepsilon=\varepsilon(k)} \tag{9}$$

where $\hbar/\tau$ is twice of $\Sigma''(\omega)$ (assumed to be k-independent, for simplicity) and $v(k)$ is the bare Fermi velocity as a function of k around the Fermi surface, which we estimate using a tight binding parameterization similar to that used previously [18]. Because

v(k) is significantly larger along the nodal direction than away from the node, the application of this equation gives a strong weighting of the nodal states to the electrical conductivity, i.e. those states strongly dominate the conductivity. While the depletion of spectral weight from pseudogapping is not modeled in our calculation, its impact diminishes approaching the node. As discussed in supplementary notes 1, it is our view that the T^* identified by resistivity slope change is not the same as T^* identified from gapping of spectral weight, though there may be connections between the two.

Here we also briefly discuss the impact of the approximation that $1/\tau$ is k-independent, i.e. taken only from the results of the measured nodal scattering rates. Previous work [19,20] as well as our own unpublished work indicates that while the normal-state scattering rates away from the node are larger than the scattering rates along the node, the temperature dependent increases of the scattering rates, as well as the frequency dependence of Σ'' are similar everywhere in the zone. Since it is this temperature dependence that dominates the scattering rates, the approximation that $1/\tau$ is k-independent is reasonable. Also, based upon the decreased weighting of the off-nodal k-states due to their velocities, any uncertainty in $1/\tau$ away from the node is not so important.

We further note that in the Boltzmann transport calculations, the relevant charge carrier density corresponds to the Fermi surface volume $1+p$, which satisfies the Luttinger theorem on the overdoped side. However, on the underdoped side Hall effect measurements[21] and quantum oscillation measurement[22] suggests that the Luttinger theorem was violated, and the relevant charge carrier density is proportional to p, which was our choice for the Drude model calculation. To the best of our knowledge, how the effective charge carrier density evolves with doping remains an unresolved issue. Nevertheless, the qualitative resemblance between our Drude results and the Boltzmann transport results implies that a significant portion of the doping-dependent temperature dependence of resistivity comes from the doping dependence of nodal quasiparticle scattering rate alone, which is essentially captured by our phenomenological form.

Supplementary Note 7. Extracting the effective temperature scales (T^*) from the resistivity curves.

Changes in curvature have often been used to extract effective temperature scales, for example the experimental resistivity data of main manuscript fig. 4e has T^* values drawn in at the scale where linearity disappears. We tried to most closely replicate the same procedure to estimate these effective scales from the simulated resistivity data of main manuscript fig. 4c. Using the same temperature window as used for the transport data we fit a line to the high temperature portion of the curves, typically extending from 300K down to approximately 250K. We then label T' or T'' as the temperature where the resistivity deviates from the linear fit by a small constant value of 7.5 **μΩcm**.

Different fit ranges as well as different thresholds for departure from linearity would yield somewhat different effective temperature scales, but the overall trend would remain the same as there is a shift from concave-up for α>0.5 to concave-down for α<0.5, with true linearity at α=0.5. It is this change in curvature that drives this kind of extraction of *T'* and *T''* to zero at α=0.5, with the resultant "v"-shaped dependence of the temperature scales on the doping phase diagram.

Supplementary Note 8. Calculating the quasiparticle residue *Z*

The quasiparticle residue *Z* or "coherent portion" of the spectral function is a measure of the overlap between a quasiparticle and a bare electron, with this value varying between 0 and 1. A Landau Fermi Liquid is, among other things, characterized by a finite *Z*. The residue *Z* can be calculated or described in a number of ways:

1) By the zero temperature occupied spectral weight $n(k)=\int_{-\infty}^{0} A(k,\omega)d\omega$, it is the strength of the step at $k=k_F$ and T=0.
2) It can be calculated as $(1-\frac{d\Sigma'}{d\omega})^{-1}|_{\omega=0,T=0}$
3) It can be calculated as the integrated weight under the spectral function (imaginary part of the Green's function) peak at T=0, $E=E_F$ and $k=k_F$. For a true Fermi liquid, the Green's function must be composed of real poles and the integrated weight should be finite. By contrast, for a non-Fermi liquid the Green's function is composed of branch cuts, and the integrated weight should be 0.

It can be analytically proven that these methods are equivalent to each other. We start with the spectral function

$$A(k,\omega) = \lim_{\delta\to 0} \frac{\Sigma''(\omega)+\delta}{(\omega-\Sigma'(\omega)-\varepsilon_k)^2+(\Sigma''(\omega)+\delta)^2}. \quad (10)$$

where δ is an infinitesimal quantity introduced for mathematical evalution. At zero temperature, we have our proposed imaginary self-energy $\Sigma''(\omega) = \omega_N(\frac{\omega}{\omega_N})^{2\alpha}$. Since it is unphysical to have the self-energy diverging at $\omega\to\infty$, we introduce a cutoff at $\frac{\omega}{\omega_N} = \pm 1$ in our model as ω_N is the normalizing frequency. As shown below, this would also allow the analytical evaluation of the real self-energy through the Kramers-Kronig relation as $\Sigma'(\omega) = -\frac{1}{\pi}\, P\int_{-\infty}^{\infty} d\omega' \frac{\Sigma''(\omega')}{\omega'-\omega} = -\frac{1}{\pi}\int_{-\omega_N}^{\omega_N} d\omega' \frac{\Sigma''(\omega')}{\omega'-\omega}$.

First, we show that method 1 gives the same result as method 3. By definition, in method 1 we have

$$n(k) = \lim_{k\to k_F^-} \int_{-\infty}^{0} \lim_{\delta\to 0} \frac{\Sigma"(\omega)+\delta}{(\omega-\Sigma'(\omega)-\varepsilon_k)^2+(\Sigma"(\omega)+\delta)^2} d\omega - \lim_{k\to k_F^+} \int_{-\infty}^{0} \lim_{\delta\to 0} \frac{\Sigma"(\omega)+\delta}{(\omega-\Sigma'(\omega)-\varepsilon_k)^2+(\Sigma"(\omega)+\delta)^2} d\omega.$$

Since that $\Sigma"(\omega)$ is an even function in our expression, $\Sigma'(\omega)$ is an odd function, and in the limit $k \to k_F$ the dispersion ε_k can be regarded as linear (i.e. $\lim_{k\to k_F^-} \varepsilon_k = -\lim_{k\to k_F^+} \varepsilon_k$), we have

$$n(k) = \lim_{k\to k_F^-} \int_{-\infty}^{0} \lim_{\delta\to 0} \frac{\Sigma"(\omega)+\delta}{(\omega-\Sigma'(\omega)-\varepsilon_k)^2+(\Sigma"(\omega)+\delta)^2} d\omega - \lim_{k\to k_F^-} \int_{0}^{\infty} \lim_{\delta\to 0} \frac{\Sigma"(-\omega)+\delta}{(-\omega-\Sigma'(-\omega)+\varepsilon_k)^2+(\Sigma"(-\omega)+\delta)^2} d(-\omega) = \lim_{k\to k_F^-} \int_{-\infty}^{\infty} \lim_{\delta\to 0} \frac{\Sigma"(\omega)+\delta}{(\omega-\Sigma'(\omega)-\varepsilon_k)^2+(\Sigma"(\omega)+\delta)^2} d\omega,$$

which is essentially the definition in method 3.

Next, we make the connections between the definition in method 3 and that in method 2. For convenience, we use dimensionless variables

$$x = \frac{\omega}{\omega_N}, \varepsilon_k = \frac{\varepsilon_k}{\omega_N}, \delta = \frac{\delta}{\omega_N}, \Sigma"(\mathrm{x}) = \frac{\Sigma"(\omega)}{\omega_N} = x^{2\alpha}, \Sigma'(\mathrm{x}) = \frac{\Sigma'(\omega)}{\omega_N}, \tag{11}$$

to rewrite the spectral function as

$$A(k,\omega) = \frac{1}{\omega_N} \lim_{\delta\to 0} \left[\frac{x^{2\alpha}}{(x-\Sigma'(x)-\varepsilon_k)^2 + (x^{2\alpha}+\delta)^2} + \frac{\delta}{(x-\Sigma'(x)-\varepsilon_k)^2 + (x^{2\alpha}+\delta)^2}\right]. \tag{12}$$

To evaluate the spectral function, we note that the second term either diverges at $x \to 0, \delta \to 0$, or approaches 0 at $x \neq 0, \delta \to 0$ (i.e. the delta function). Define

$$\lim_{\delta\to 0} \frac{\delta}{(x-\Sigma'(x)-\varepsilon_k)^2+(x^{2\alpha}+\delta)^2} = \lim_{\delta\to 0} S_\delta(x). \tag{13}$$

If

$$\int_A^B dx F(x) \lim_{\delta\to 0} S_\delta(x) = \pi Z F(C) \tag{14}$$

where A, B, C are constants and $F(x)$ is an infinitely differentiable function, then

$$\lim_{\delta\to 0} S_\delta(x) = \pi Z \delta(x-C), \tag{15}$$

which essentially defines the quasiparticle residue Z with the integral of spectral function as in method 3. For the case of $2\alpha > 1$, rewrite

$$\int_B^A dxF(x) \lim_{\delta\to 0} S_\delta(x) = \lim_{\delta\to 0} \int_B^A d\frac{x}{\delta} F(x) \frac{1}{(\frac{x}{\delta}-\frac{\Sigma'(x)}{\delta}-\varepsilon_k/\delta)^2+(\frac{x^{2\alpha}}{\delta}+1)^2}), \tag{16}$$

and let $y = x/\delta$, $k = k_F$ (i.e. $\varepsilon_k = 0$), we have

$$\int_B^A dxF(x) \lim_{\delta\to 0} S_\delta(x) = \lim_{\delta\to 0} \int_{B/\delta}^{A/\delta} dyF(y\delta) \frac{1}{y^2(1-\frac{\Sigma'(y\delta)}{y\delta})^2+(y^{2\alpha}\delta^{2\alpha-1}+1)^2}). \tag{17}$$

Using supplementary equation 14 we have

$$\lim_{\delta\to 0} \int_{-\infty}^{+\infty} dyF(0) \frac{1}{y^2(1-\frac{\Sigma'(y\delta)}{y\delta})^2+(y^{2\alpha}\delta^{2\alpha-1}+1)^2}) = \pi Z F(0). \tag{18}$$

Since $\int_{-\infty}^{\infty} dy \frac{1}{1+y^2} = \pi$, therefore

$$Z = \lim_{\delta\to 0} \left(1-\frac{\Sigma'(y\delta)}{y\delta}\right)^{-1},$$

which is the definition as in method 2. On the other hand, in the $2\alpha < 1$ case, with $z = x^{2\alpha}/\delta$ we have

$$\int_B^A dxF(x) \lim_{\delta\to 0} S_\delta(x) = \lim_{\delta\to 0} \int_{B^{2\alpha}/\delta}^{A^{2\alpha}/\delta} \frac{dz}{2\alpha} F(\delta^{1/2\alpha} z^{1/2\alpha}) \frac{\delta^{\frac{1}{2\alpha}-1} z^{\frac{1}{2\alpha}-1}}{(\delta^{\frac{1}{2\alpha}-1} z^{1/2\alpha})Z^2+(z+1)^2}). \tag{19}$$

Since $\frac{1}{2\alpha} > 1$, the limit vanishes, $Z = 0$, so there is no quasiparticle contribution to the spectral density.

Now we have shown the three methods being equivalent to each other, we can calculate Z with the expression

$$Z = \left(1-\frac{d\Sigma'}{d\omega}\right)|_{\omega=0,T=0}, \tag{20}$$

in which the real part of self-energy can be obtained with the Kramers-Kronig relation

$$\Sigma'(x) = -\frac{1}{\pi}\int_{-1}^{1} dy \frac{\Sigma''(y)}{y-x} = -\frac{1}{\pi}\int_{-1}^{1} dy \frac{y^{2\alpha}}{y-x}. \tag{21}$$

To approach the $\omega \to 0$ limit, we have

$$\Sigma'(\delta) = -\frac{1}{\pi}\int_{-1}^{1} \frac{y^{2\alpha}}{y-\delta} dy = -\frac{1}{\pi}\int_{-\delta}^{1-\delta} \frac{(y+\delta)^{2\alpha}}{y} dy + \frac{1}{\pi}\int_{\delta}^{1+\delta} \frac{(y-\delta)^{2\alpha}}{y} dy \tag{22}$$

Expand the numerator to first order in δ as

$$(y \pm \delta)^{2\alpha} \approx y^{2\alpha} \pm 2\alpha y^{2\alpha-1}\delta, \tag{23}$$

and we find

$$\begin{aligned}\Sigma'(\delta) &= -\frac{1}{\pi}\int_{-\delta}^{1-\delta}\frac{y^{2\alpha}}{y}dy + \frac{1}{\pi}\int_{\delta}^{1+\delta}\frac{y^{2\alpha}}{y}dy + \frac{4\alpha\delta}{\pi}\int_0^1\frac{y^{2\alpha-1}}{y}dy \\ &= -\frac{1}{\pi}\int_{-\delta}^{\delta} y^{2\alpha-1}\,dy + \frac{1}{\pi}\int_{1-\delta}^{1+\delta} y^{2\alpha-1}dy + \frac{4\alpha\delta}{\pi}\int_0^1\frac{y^{2\alpha-1}}{y}dy \\ &= -\frac{1}{\pi}\left[0 - \frac{y^{2\alpha}}{2\alpha}\Big|_{1-\delta}^{1+\delta} - 4\alpha\delta\frac{y^{2\alpha-1}}{2\alpha-1}\Big|_0^1\right] = \frac{\delta}{\pi}[2 - 4\alpha\frac{y^{2\alpha-1}}{2\alpha-1}\Big|_0^1.]\end{aligned} \tag{24}$$

In the case $2\alpha - 1 < 0$, $\frac{y^{2\alpha-1}}{2\alpha-1}\Big|_0^1$ diverges to infinity. Therefore

$$Z = \lim_{\delta\to 0}\left(1 - \frac{\Sigma'(\delta)}{\delta}\right)^{-1} = 0. \tag{25}$$

On the other hand, when $2\alpha - 1 > 0$,

$$Z = \lim_{\delta\to 0}\left(1 - \frac{\Sigma'(\delta)}{\delta}\right)^{-1} = (1 + \frac{2}{\pi}\left(\frac{2\alpha}{2\alpha-1} - 1\right))^{-1} = \frac{(2\alpha-1)\pi}{2+(2\alpha-1)\pi}. \tag{26}$$

These are the analytical forms plotted in main manuscript Fig 5. Please note that while the exact value of Z in the $2\alpha - 1 > 0$ case depends on the cutoff introduced in our model as in supplementary equation 19, for the $2\alpha - 1 < 0$ case Z is always 0.

In parallel to the analytical derivation, numerical simulations have been performed with the Wolfram Mathematica software to independently determine the quasiparticle residue Z with these methods. In particular, with the definition in supplementary equation 21, the Mathematica software was able to find an analytical evaluation of Σ' in the form of Hypergeometric functions:

$$\Sigma'(\omega,\alpha) = -\frac{1}{\pi}Sign[\omega]Abs\left[\frac{i}{\omega(1+2\alpha)}\left(-\pi\omega^{1+2\alpha} - 2\alpha\pi\omega^{1+2\alpha} + iHypergeomtric2F1\left[1,1+2\alpha,2(1+\alpha),-\frac{1}{\omega}\right] + iHypergeomtric2F1\left[1,1+2\alpha,2(1+\alpha),\frac{1}{\omega}\right]\right)\right], \tag{27}$$

which can be used for numerical derivative in method 2 and numerical integral in method 1 and 3. As shown in supplementary fig. 5, the numerical simulation results converge to the analytical solution in supplementary equation 25 and 26 with an increasing precision. It shows that even though at finite temperatures the PLL spectral function gives clear spectral peaks in ARPES and in the Drude weight for α<0.5, at the zero temperature limit, these peaks would have residue *Z=0* at *T=0* and

must be a true non-Fermi liquid metal. For α>0.5 there are true quasiparticle peaks with finite residue, though these should still not be classified as a traditional Fermi liquid because many of the properties of a Fermi liquid are not maintained.

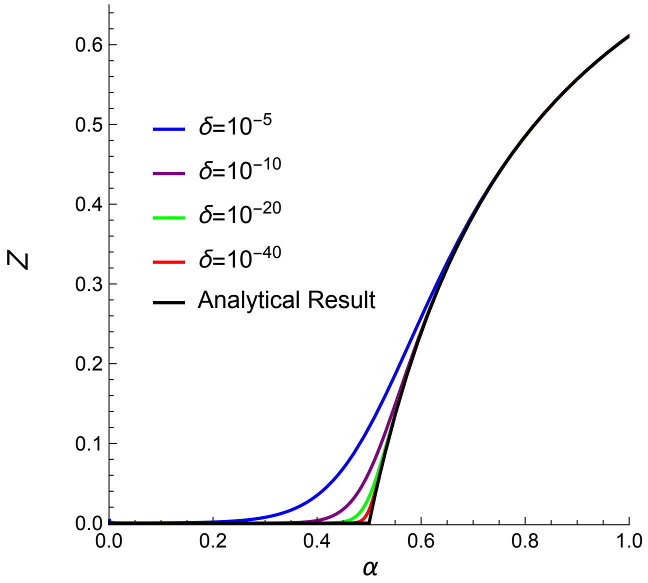

Figure 5. Numerical simulation of the quasiparticle residue Z. With an increasing precision, the simulation converges to the analytical result in supplementary equation 25 and 26.

Supplementary Note 9. Calculated optical conductivity and comparison to measurements

Many measurements of the optical conductivity of the cuprates have been carried out, with generally anomalous results that do not match findings of other more standard electronic materials [23]. Fig 5c of the main text shows T=300K measured optical conductivity of Bi2212 as a function of doping, extracted from Hwang et al.[24]. The data show an anomalous low-energy peak that that weakens in intensity with increased underdoping, indicating some kind of reduced coherence of the electronic states. This is in contrast to the expectations of a Fermi Liquid, which would maintain the area under the “Drude” peak with increased broadening or correlations:

Fig. 5b of the main text shows the calculated normal state optical conductivity using the PLL spectral function. Optical conductivity is typically calculated using the following equation[25]:

$$\sigma = \sigma_1 + i\sigma_2 = \frac{ne^2}{m}\frac{i}{i\tau_{optcal}^{-1}(\omega)+\omega(1+\lambda(\omega))} \tag{28}$$

where the optical scattering rate $\tau_{optical}^{-1}(\omega)$ and $\omega\lambda(\omega)$ is related through the Kramers-Kronig relation. For simplicity, here we related these two terms to twice of our imaginary and real parts of self-energy respectively as in equation (1). Different dopings are determined just as in the main paper, with color coding set to match those used for panel c. No parameters at all were adjusted to produce the simulated data of

fig. 5b: the α values were determined by the linear fit to doping from fig. 1c and the other parameters were set to their nominal values, i.e. Γ_0=0 meV, β=π, λ=0.5 and ω_N=0.5 eV. No scaling of either of the axes was carried out. Even though our simulations are only based on self-energies along the nodal direction instead of over the whole Brillouin zone, they capture the main characteristics of the optical conductivity data. We note however, the agreement becomes slightly less satisfactory on the underdoped side in terms of magnitudes. It is likely that a more advanced and complete model is needed to directly connect the ARPES and optical conductivity results in this region.

Supplementary Note 10. Fermi-liquid-like resistivity and relaxation rates in underdoped cuprates.

There are a few reports of upwards rather than downwards curvature of resistivity vs. temperature in the low temperature regime of underdoped samples [26,27,28], which at first glance seems inconsistent with the PLL self-energy. Additionally, right above the temperature range in which the upturn manifests or should have manifested without the superconducting transition, there have been reports of a T squared resistivity in the pseudogap phase [26, 28], which has been interpreted as signaling a Fermi Liquid-like regime. While neither of these effects seem consistent with the PLL phenomenology at underdoping, considering both of them together can help explain the apparent inconsistencies.

The first effect is the strong upturn in the resistivity at low temperatures that becomes especially obvious when the superconductivity is quenched by a magnetic field, i.e. the Ando-Boebinger effect (see for example fig. 1a in ref 29). While this upturn is not understood, it can to zeroth order be considered to be some sort of localization physics and has been modeled as a log(1/T) behavior at low temperatures [29]. Unfortunately, many aspects of this phenomenology are inconsistent with a localization phenomenon – for example $k_F l$ remains relatively large. Here we note that superimposing this log(1/T) behavior with the PLL s-shaped behavior can give a T^2-like dependence to the resistivity, though deconvolving these two terms in transport would be very difficult, especially since it is not known how high in temperature the log (1/T) upturn survives (efforts to extract power law scattering from transport have so far ignored this log(1/T) at higher temperatures). In contrast to transport, ARPES has the advantage that the log(1/T) localization physics is not expected to impact the spectral function at all, so is a more direct way to access the details of the true electronic scattering rates.

We now turn to the relaxation rates. In ref. [26], it was proposed from optical conductivity data that the relaxation rate of underdoped cuprates follow a $\zeta^2=\omega^2+(\beta k_B T)^2$ dependence. This seems to be at odds with our PLL form as this would imply a PLL parameter α =1 even for underdoped samples.. However, we argue that the data from [26] fit better with a Power Law Liquid form with α significantly less than 1 over a larger energy and temperature range, as shown in supplementary Fig.

6. In particular, we find that α=0.56 for Bi2201, α=0.65 for YBCO, and α=0.70 for Hg1201 – values inconsistent with a Fermi Liquid but consistent with our Power Law Liquid phenomenology.

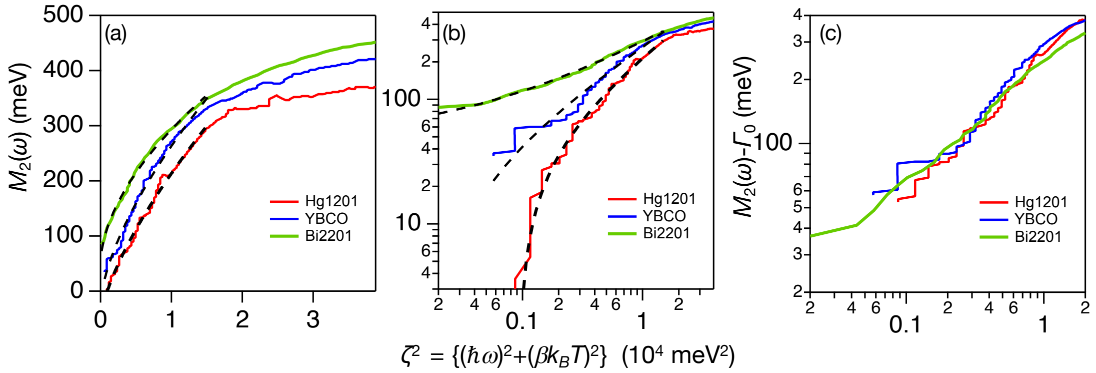

Figure 6. Imaginary part of the optical memory function for three underdoped samples, extracted from Fig 6 of Mirzaei et al.[26]. The curves of (a) are the approximate average of each of the many temperature plots of ref.[26] Fig 6, while (b) is the same data plotted in log-log format and (c) is the same data after a constant offset Γ_0 has been removed so as to give the best fit to the form $M_2(\omega)=\Gamma_0+c(\zeta^2)^\alpha$ (dashed lines in panels a and b). If these materials were governed by Fermi liquid scattering, the data of (c) would be linear with unity slope (α=1). Instead, we find that the slopes are sublinear, with α=0.56 for Bi2201, α=0.65 for YBCO, and α=0.70 for Hg1201. Also unusual are the negative offset values Γ_0=-51 meV for Hg1201 and Γ_0=-22 meV for YBCO (Bi2201 has positive Γ_0=50 meV).

Such a power law dependence might also be evident in Fig. 5 of ref. [26]. Even though the inset gave the impression that all the curves are linear in ζ^2, a fan-shape $M_2(\omega)$ can be seen once we view the dataset as a whole, from low temperature to high temperature. Such behavior mimics our data in main manuscript Fig. 1. To illustrate this point, we use the underdoped T_c = 63 K sample data as an example and fit it with both the PLL form and the traditional Fermi liquid form for all temperatures. As shown in supplementary Fig. 7, the PLL form provides a much more accurate description of the phenomenology than the Fermi liquid form, indicating that we advanced beyond the work in ref. [26].

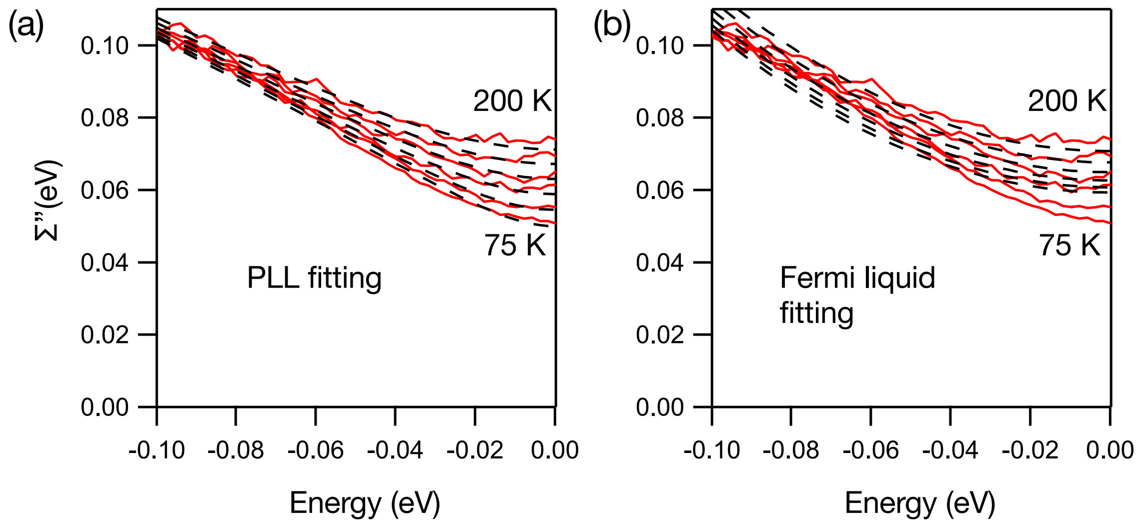

Figure 7. **Comparison of PLL fitting and Fermi liquid fitting of ARPES data.** Imaginary self-energy of the underdoped T_c = 63 K sample from 75 K to 225 K, fit to a) the power law liquid phenomenological form and b) the traditional Fermi liquid form. The variance of a) is 6 times smaller than that of b).

1 Koralek, J.D. et al. Laser Based Angle-Resolved Photoemission, the Sudden Approximation, and Quasiparticle-Like Spectral Peaks in $Bi_2Sr_2CaCu_2O_{8+\delta}$. Phys. Rev. Lett. 96, 017005 (2006)

2 Reber, T.J., Plumb, N.C., Dessau, D.S., Effects, determination, and correction of count rate nonlinearity in multi-channel analog electron detectors. Rev. Sci. Instrum. **85**, 043907 (2014)

3 Hüfner S., Hossain M. A., Damascelli A., and Sawatzky G. A., Two gaps make a high-temperature superconductor? Reports on Progress in Physics 71 (2008), ISSN 0034-4885.

4 Wang, Y.Y. et al. Field-enhanced diamagnetism in the pseudogap state of the cuprate $Bi_2Sr_2CaCu_2O_{8+d}$ superconductor in an intense magnetic field. Phys. Rev. Lett. 95, 247002 (2005).

5 Dubroka, A. et al., Evidence of a Precursor Superconducting Phase at Temperatures as High as 180 K in $RBa_2Cu_3O_{7-d}$ (R =Y; Gd; Eu) Superconducting Crystals from Infrared Spectroscopy. Phys. Rev. Lett 106, 047006 (2011)

6 Reber T.J. et al, Prepairing and the "filling" gap in the cuprates from the tomographic density of states. Phys. Rev. B 87, 060506 (2013)

7 Tanaka, K. et al. Distinct Fermi-momentum-dependent energy gaps in deeply underdoped Bi2212. Science 314, 1910–1913 (2006).

8 Kondo, T. et al. Competition between the pseudogap and superconductivity in the high-Tc copper oxides. Nature 457, 296–300 (2009).

9 Hussey N.E., Phenomenology of the normal state in-plane transport properties of high-T_c cuprates. J. Phys.: Condens. Matter 20, 123201 (2008)

10 Timusk, T., Statt B., The pseudogap in high temperature superconductors: an experimental survey. Rep. Prog. Phys. 62, 61 (1999)

11 Berthod, C. et al. Non-Drude universal scaling laws for the optical response of local Fermi liquids. Phys Rev B **87**, 115109 (2013)

12 Abrahams, E. and Varma, C. M, What angle-resolved photoemission experiments tell about the microscopic theory for high-temperature superconductors. Proc. Natl. Acad. Sci. U.S.A. **97**,5714 (2000)

13 Varma, C. M and Abrahams, E., Effective Lorentz Force due to Small-Angle Impurity Scattering: Magnetotransport in High-*Tc* Superconductors. Phys. Rev. Lett. **86**, 4652 (2001)

14 Millis, A. J. and Drew, H. D., Quasiparticles in High Temperature Superconductors: Consistency of angle-resolved photoemission and optical conductivity. Phys. Rev. B **67**, 214517 (2003)

15 Reber, T.J. et al., The Non-Quasiparticle Nature of Fermi Arcs in Cuprate High-T_C Superconductors Nature Physics **8**, 606–610 (2012)

16 Parham, S., Reber, T.J., Cao, Y., Waugh, J.A., Gu, G., Dessau, D.S., Effects of Fe Impurities on the Gap and Electronic Scattering Rates in the High-Tc Superconductor $Bi_{2.1}Sr_{1.9}Ca(Cu_{1-y}Fe_y)_2O_x$. PRB 87, 104501 (2013)

17 Ashcroft, N.W. and Mermin N.D., Solid State Physics Saunders College (1976)

18 Markiewicz R.S. et al., One-band tight-binding model parametrization of the high-T_c cuprates including the effect of k_z dispersion. PRB 72, 054519 (2005)

19 T. Valla Temperature Dependent Scattering Rates at the Fermi Surface of Optimally Doped $Bi_2Sr_2CaCu_2O_{8+d}$. PRL 85, 828 (2000)

20 Kondo, T. et al., Electrical resistivity and scattering processes in $(Bi,Pb)_2(Sr,La)_2CuO_{6+d}$ studied by angle-resolved photoemission spectroscopy. Phys Rev B 74, 224511 (2006)

21 Ong, N. P. et al., Hall effect in $La_{2-x}Sr_xCuO_4$ Implications for the electronic structure in the normal state. Phys Rev B 35, 8807 (1987)

22 Doiron-Leyraud, N. et al., Quantum oscillation and the Fermi surface in an underdoped T_c superconductor *Nature* **447**, 565-568 (2007).

23 Basov, D.N., & Timusk, T. Electrodynamics of high-*Tc* superconductors Review of Modern Physics, **77**, 721 (2005)

24 Hwang, J. et al, Marginal Fermi liquid analysis of 300 K reflectance of $Bi_2Sr_2CaCu_2O_{8+\delta}$, Phys. Rev. B **69**, 094520 (2004)

25 Carbotte, J.P., Schachinger, E. and Hwang, J., Boson structures in the relation between optical conductivity and quasiparticle dynamics, Phys. Rev. B **71**, 054506 (2005)

26 Mirzaei S.I. et al., Evidence for a Fermi liquid in the pseudogap phase of high-T_c cuprates Proceedings of the National Academy of Sciences 110, 5774-5778 (2013)

27 Barišić, N., et al. Universal sheet resistance and revised phase diagram of the cuprate high-temperature superconductors PNAS 110 (30), 12235-12240 (2013)

28 Cooper, R. A., Anomalous Criticality in the Electrical Resistivity of $La_{2-x}Sr_xCuO_4$. Science 323, 5914, 603-607 (2009). [DOI:10.1126/science.1165015]

29 Boebinger, G.S. et al., Insulator-to-Metal Crossover in the Normal State of $La_{2-2x}Sr_xCuO_4$ Near Optimum Doping. Phys. Rev. Lett. 77, 5417 (1996)
